# Does Gender Make a Difference in Deception? The Effect of Transcranial Direct Current Stimulation Over Dorsolateral Prefrontal Cortex

**DOI:** 10.3389/fpsyg.2018.01321

**Published:** 2018-08-20

**Authors:** Mei Gao, Xiaolan Yang, Jinchuan Shi, Yiyang Lin, Shu Chen

**Affiliations:** ^1^College of Economics, Zhejiang University, Hangzhou, China; ^2^School of Business and Management, Shanghai International Studies University, Shanghai, China; ^3^Academy of Financial Research, Zhejiang University, Hangzhou, China; ^4^Interdisciplinary Center for Social Sciences, Zhejiang University, Hangzhou, China

**Keywords:** deception, dorsolateral prefrontal cortex, transcranial direct current stimulation, gender difference, cheap talk

## Abstract

Neuroimaging studies have indicated a correlation between dorsolateral prefrontal cortex (DLPFC) activity and deceptive behavior. We applied a transcranial direct current stimulation (tDCS) device to modulate the activity of subjects’ DLPFCs. Causal evidence of the neural mechanism of deception was obtained. We used a between-subject design in a signaling framework of deception, in which only the sender knew the associated payoffs of two options. The sender could freely choose to convey the truth or not, knowing that the receiver would never know the actual payment information. We found that males were more honest than females in the sham stimulation treatment, while such gender difference disappeared in the right anodal/left cathodal stimulation treatment, because modulating the activity of the DLPFC using right anodal/left cathodal tDCS only significantly decreased female subjects’ deception.

## Introduction

Deception is a complex human behavior that is prevalent in finance, politics and interpersonal relationships. It is widespread in various sectors of society and has important economic consequences (Gachter and Schulz, 2016). Numerous fraud scandals in recent years have greatly damaged the economy and the stability of financial markets (Abrantes-Metz et al., 2012; Sapienza and Zingales, 2012). In a situation involving asymmetric information, businessmen, politicians and others may deliberately take advantage of private information to deceitfully improve their self-earnings (Gneezy, 2005; Clotsfigueras et al., 2015). Therefore, determining what maintains human honesty and how to prevent deceptive behavior, especially in the economy, is a fundamental problem.

In reality, most fraudsters in social economies expect to increase their profits by a series of lies that lead to the decreasing earnings of others. It must be emphasized that honest choices are always associated with conflicts between self-interest and others’ interests. It’s obvious that financial honesty concerns moral norms that help us to resist the temptation of making more money by behaving dishonestly (Villeval, 2014). Why people sometime could sacrifice monetary payoffs and be truthful? The moral conflicts elicited by dishonest gain play a significant role in human deceptive behavior (Mead et al., 2009), while little is known about the neural process of human when resolving the conflict between honesty and monetary gain.

Some studies relied on instructed-lying paradigms show that deception requires the host of executive functions as people need to inhibit the disclosure of the truth to make deceptive responses (Hu et al., 2011). Conflict related deception involves executive function in dorsalateral prefrontal cortex (DLPFC), ventralateral prefrontal cortex (VLPFC), medial frontal cortex (MFC) and anterior cingulate cortex (ACC) (Ganis et al., 2003; Abe et al., 2006). As studies using instructed-lying paradigms typically examine deception ability rather than deceptive behavior (Sip et al., 2008), other studies pay attention to the neural mechanisms underlying spontaneous deception (Greene and Paxton, 2009; Ding et al., 2013). Wu et al. (2009) found a more positive P300 amplitude triggered by self-determined response than that triggered by forced responses. What’s more, the N2, which indicates subjects’ conflict detection, was more negative elicited by deceptive response than that elicited by honest response. It seems that the brain response of both instructed deception and spontaneous deception is conflict related. However, in the most studies of spontaneous deception, subjects’ gains were not directly associated with their dishonest or honest decisions. That is, they didn’t face the moral trade-off between deceptive behavior and self-interest. Only one study has investigated spontaneous deception considering the moral conflict between honesty and self-interest (Greene and Paxton, 2009). In a simple game asking subjects undergoing functional magnetic resonance imaging (fMRI) to self-report the accuracy of coin-flip predictions, they found that increased activity in the DLPFC was closely associated with dishonest subjects’ decisions compared to subjects behaving honestly, both when telling lies and occasionally telling truth. As neuroimaging studies can only demonstrate a correlation between the activity of certain cortex areas and deceptive behavior, the causal effect remains unknown. Thus, the neural basis of deceptive behavior in DLPFC remains unexplored especially in the setting involving moral conflict between honesty and personal gain.

Increasingly, brain stimulation techniques are being used in research (Li et al., 2017; Maréchal et al., 2017; Wang et al., 2017). Such techniques can enable direct observations of how modulating the activity of the DLPFC affects subjects’ deceptive behavior. Maréchal et al. (2017) tested subjects’ honest behavior using a die-rolling task with transcranial direct current stimulation (tDCS) over the right dorsolateral prefrontal cortex (rDLPFC). They showed that honesty was enhanced after anodal stimulation of the rDLPFC. To the best of our knowledge, this was the first paper to demonstrate that honesty can be strengthened through non-invasive stimulation of the DLPFC. Further research using different experimental paradigms is needed to excavate the neural mechanism of honest behavior robustly.

Some studies of cheating behavior have adopted a “cheap talk sender–receiver” game (e.g., Gneezy, 2005; Zhu et al., 2014), in which only the sender knew the associated payoffs of two options and freely chose to convey the truth or not, knowing that the receiver would never know the actual payment information. This game contains the conflict between self-interest and honesty.

Unlike the die-rolling task adopted by Maréchal et al. (2017), measuring aggregate-level of honesty, the cheap talk sender–receiver game enables us to utilize individual-level data to analyze the deceptive behavior. Except that we collected individual-level data of deception, we could clearly justify whether subjects made an honest decision or not in our study, while in the die-rolling task, there were some probability that subjects reported the profit-maximizing outcome because that was the actual outcome. Obviously, it was not the case that we were intend to investigate, where subjects needed to decide whether to behave dishonestly for self-interest or not. What’s more, though subjects were anonymous in both experiments, subjects knew that their deceptive behavior could be observed by experimenters only in the cheap-talk sender-receiver game. Therefore, the psychic cost of deception is different in the two experiments. Although the classical die-rolling experimental study conducted by Fischbacher and Follmiheusi (2013) showed that the reported distribution was not significantly changed when the remainder was given to another subject instead of being kept by the experimenter, the two experiments are still different in paradigm itself.

To investigate the effect of tDCS on individuals’ deceptive behavior related to the moral conflict between self-interest and others’ interests, our experiment used a cheap talk sender–receiver game in which senders had private information about the real allocation of money between themselves and their paired receivers and then decided to send an honest/dishonest message about the allocation to receivers. We adopted a between-subject design to test whether various tDCS treatments changed subjects’ honesty by comparing the subjects’ deceptive behavior among different stimulation treatments. Our goal was to find a causal relationship between DLPFC and deceptive behavior, and to compare the exact effect of different stimulations on the honesty of subjects when there are interest conflicts between senders and receivers and when there are no interest conflicts.

Since a sender might choose to tell the truth strategically if he/she expected the receiver not to follow his/her message, the cheap talk sender–receiver game in our experiment might confound honesty with strategic motives. An additional questionnaire was also conducted to directly verify the senders’ strategic consideration.

## Materials and Methods

### Subjects

Hundred and eighty subjects were recruited from different majors at Zhejiang University via an advertisement posted on the school bulletin board system. Subjects were grouped in pairs and randomly assigned the role of sender or receiver. Ninety subjects (46 females, mean age = 21.4 ± 2.07 years, all right-handed) who acted as senders, were randomly assigned to three treatment groups: right anodal/left cathodal stimulation (*n* = 30), left anodal/right cathodal stimulation (*n* = 30) or sham (*n* = 30) treatment. The experiment lasted around 40 min, and the average payment of the subjects was CNY 22.88 (approximately 3.46 dollars). To learn the senders’ beliefs regarding the reaction of the receivers, 57 senders (29 females, mean age = 21.02 ± 2.2 years, all right-handed) were asked to sequentially complete a questionnaire. All of the subjects gave written informed consent, and the study was approved by the Zhejiang University ethics committee before the start of the experiment. No subjects reported any adverse side effects regarding pain on the scalp or headaches after the experiment.

### Transcranial Direct Current Stimulation

Transcranial direct current stimulation (tDCS), a non-invasive brain stimulation technique, was delivered by a battery-driven multichannel non-invasive wireless neurostimulator (Starlab, Spain). A constant 2-mA current flow lasting for 20 min with 30 s of ramp up and down was applied via a pair of saline-soaked sponge electrodes (5 cm × 7 cm) fixed on the scalp of the participant with a rubber belt. tDCS facilitates neural excitability depending on electrode polarity. The anodal electrode enhances cortical excitability while the cathodal electrode weakens it (Nitsche and Paulus, 2000). As in the study by Gandiga et al. (2006), the current delivered in the sham stimulation treatment only lasted for 30 s once it reached 2 mA. This short-lived but perceptible stimulation was designed to make the subjects feel as if they had received the true stimulation treatment.

**FIGURE 1:**
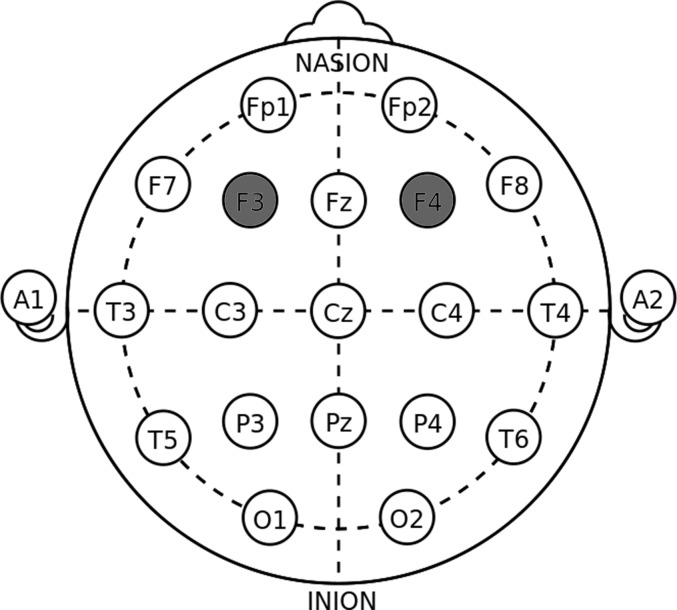
Electrode placements in DLPFC stimulations.

Electrodes placed over F3 and F4 can effectively influence the DLPFC area (Fecteau et al., 2007a,b; Boggio et al., 2009). As shown in **Figure 1**, the anodal (cathodal) electrode was placed over the right F4 and the cathodal (anodal) electrode was placed over the left F3 in the right anodal/left cathodal (left anodal/right cathodal) treatment based on the International 10-20 System for electrode placement.

### Experimental Design

The cheap talk sender–receiver game is a two-player communication game in which one player (the sender) has private information and the other (the receiver) makes the final allocation decision (Gneezy, 2005; Zhu et al., 2014). In the experiment, the subjects were grouped in pairs and randomly assigned the role of sender or receiver. A screen was set up to separate senders and receivers, so that the sender and the receiver in a pair never meet. The game was composed of 12 trials. For each group, only the subject who played the role of the sender was informed about the monetary payoffs of two options, A and B, in each trial. The sender had to send one of two messages to the other subject in the role of receiver:

Message 1: “Option A will earn you more money than option B.”
Message 2: “Option B will earn you more money than option A.”

After receiving the message sent by the sender, the receiver chose the option to be carried out. Crucially, all senders knew that receivers would never be informed of the payoffs associated with each option. Therefore, they could choose either the honest or dishonest message. At the end of the game, we randomly chose one of the 12 trials to determine the real payoff for the subjects.

The monetary consequences varying across trials are displayed in **Table 1**, following Zhu et al. (2014). For instance, option A corresponds to CNY15 to the sender and CNY5 to the receiver, and option B corresponds to CNY5 to the sender and CNY15 to the receiver in Trial 1. It is obvious that the sender’s honest choice, that is, sending message 2, “Option B will earn you more money than option A,” to the receiver, will damage his/her own payoff. Thus, there is a conflict between self-interest and others’ interests, as an honest message will result in the sender allocating less money to himself/herself but more to the receiver. These trials are referred to as “conflict trials” (C). There are also “no-conflict trials” (NC), in which the sender’s interest is aligned with the receiver’s interest (Trials 5 and 9).

**Table 1:** The cheap talk sender–receiver game.

Trial number	Option A		Option B		Interest conflict	Honest message
	Self	Other	Self	Other		
1	15	5	5	15	C	2
2	10	5	5	20	C	2
3	6	5	10	4.99	C	1
4	5	10	10	5	C	1
5	8	10	10	12	NC	2
6	6	5	5	6	C	2
7	5	20	20	5	C	1
8	6	5	5	15	C	2
9	10	6	10	5	NC	1
10	10	12	12	10	C	1
11	5	10	6	5	C	1
12	10	4.99	4	5	C	2


### Experimental Procedure

At the beginning of the experiment, subjects were randomly assigned to a sender or a receiver and asked to sign the written informed consent form. Then, the researchers placed tDCS devices on the sender’s head for a 20-min stimulation and told them to seat themselves comfortably and relax. The devices were taken away when the stimulation ended. After a public reading of experimental instructions, the experiment was conducted by the software z-Tree (Fischbacher, 2007). Trials were presented one by one. At the end of the experiment, the computer randomly selected one trial as the payoff. The final payments were the combination of a show-up fee and the payoffs in the selected trials according to receivers’ decisions.

In the experiment, we collected each subject’s percentage of honest choices and “amount given” in conflict trials and in no-conflict trials. Following Zhu et al. (2014) we adopted a measure of honesty called “amount given,” which was defined as the amount that senders were willing to allocate to receivers according to the message sent by senders. Taking Trial 1 as an example, if the sender tells the truth (message 2), then the amount given is CNY15 in Option B; while if the sender lies (message 1), it is CNY5 in Option A.

## Results

### Effect of tDCS on Deceptive Behavior

**FIGURE 2:**
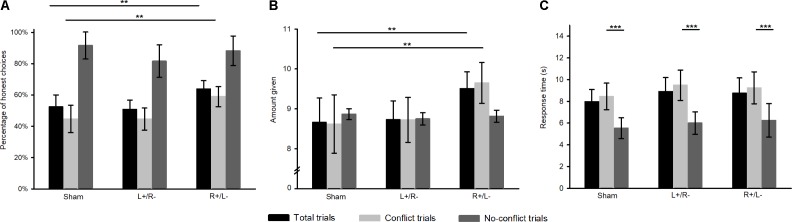
Deceptive behavior in different trials and treatments after the stimulations. Error bars indicate 95% confidence intervals. **(A)** Average percentage of honest choices. **(B)** Average amount given. **(C)** Average response time. Significance level: ^∗∗∗^ 1 percent, ^∗∗^ 5 percent, ^∗^ 10 percent.

To test whether different tDCS treatments changed subjects’ deceptive behavior, we compared the percentage of honest and dishonest choices after the treatment. Deception was substantial in the sham stimulation treatment group, in which senders cheated in half of the trials (**Figure 2A**). However, the deceptive behavior was concentrated in the conflict trials, and in no-conflict trials, the cheating proportion was only 8.3%.

A 2 conflict condition (conflict trials vs. no-conflict trials) × 3 stimulation type (right anodal/left cathodal stimulation vs. left anodal/right cathodal stimulation vs. sham stimulation) ANOVA on the average percentage of honest choices revealed a significant main effect of conflict condition, *F*_1,87_ = 114.675, *p* < 0.000, with subjects choosing less honest choices in conflict trials (mean = 49.4%) compared to no-conflict trials (mean = 87.2%). Though the interaction of conflict condition and stimulation type was not significant, *F*_1,87_ = 2.102, *p* = 0.128, a main effect of stimulation type was found, *F*_1,87_ = 3.389, *p* = 0.038. The average percentage of honest choices was higher after the right anodal/left cathodal stimulation in conflict trials (R+/L- mean = 59%, sham mean = 44.7%, *p* = 0.021), but there was no significant difference after the left anodal/right cathodal stimulation in conflict trials (L+/R- mean = 44.7%, sham mean = 44.7%, *p* > 0.1). However, tDCS had little effect on senders’ deceptive behavior in no-conflict trials no matter what type of stimulation was used. In other words, right anodal/left cathodal stimulation made senders more honest only when they had to resolve the trade-off between self-interest and honesty.

A 2 conflict condition (conflict trials vs. no-conflict trials) × 3 stimulation type (right anodal/left cathodal stimulation vs. left anodal/right cathodal stimulation vs. sham stimulation) ANOVA on the amount given revealed a significant main effect of the interaction of conflict condition and stimulation type was found, *F*_2,87_ = 3.361, *p* = 0.039. Similarly, **Figure 2B** shows that senders were willing to give more to receivers after right anodal/left cathodal tDCS only in the conflict trials (R+/L- mean = 9.649, sham mean = 8.622, *p* = 0.05), whereas the left anodal/right cathodal tDCS had no influence on the amounts given to receivers (L+/R- mean = 8.725, sham mean = 8.622, *p* > 0.1). Senders were more honest after the right anodal/left cathodal tDCS of the DLPFC, especially in the conflict trials.

**Figure 2C** shows the response times for senders’ honest and dishonest decisions, revealing the different behavioral patterns between conflict trials and no-conflict trials. According to the 2 conflict condition (conflict trials vs. no-conflict trials) × 3 stimulation type (right anodal/left cathodal stimulation vs. left anodal/right cathodal stimulation vs. sham stimulation) ANOVA on the response times, senders spent more time choosing the message to send in the conflict trials than in the no-conflict trials regardless of the stimulation type (conflict trials = 9.061, no-conflict trials = 5.928, *p* < 0.000). In light of the main effect of stimulation type, there was no significant difference in response time among three stimulation types (*F*_2,87_ = 0.563, *p* > 0.1).

### Gender Difference

**FIGURE 3:**
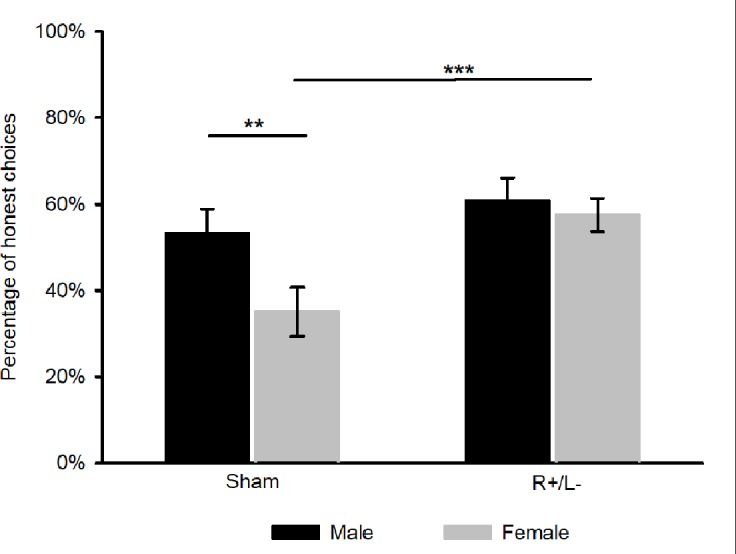
Average percentage of honest choices in conflict trials after the sham and right anodal/left cathodal stimulations over DLPFC for males and females, respectively. Error bars indicate 95% confidence intervals. Significance level: ^∗∗∗^ 1 percent, ^∗∗^ 5 percent, ^∗^ 10 percent.

As **Figure 3** shows, for females, the average percentage of honest choices was higher after the right anodal/left cathodal stimulation in the conflict trials (R+/L- mean = 57.5%, sham mean = 35%, *t_1,28_* = 3.38, *p* = 0.002), while for males, the effect of tDCS was not significant (R+/L- mean = 60.7%, sham mean = 53.1%, *t_1,28_* = 0.97, *p* = 0.341). To further determine the gender difference in deceptive behavior, we applied a two-way ANOVA with the percentage of honest choices in the conflict trials as the dependent variable, while gender and stimulation type served as independent variables. We found that males were more likely to make honest choices than females in the conflict trials in the sham stimulation treatment (males: mean = 53.1%, females: mean = 35%, *p* = 0.014). However, no significant difference in the percentage of honest choices between males and females was observed after the right anodal/left cathodal stimulation in the conflict trials (males: mean = 60.7%, females: mean = 57.5%, *p* = 0.656). Only females seemed to be altered by tDCS and became more honest after the right anodal/left cathodal stimulation (females: R+/L- mean = 57.5%, sham mean = 35%, *p* = 0.007; males: R+/L- mean = 60.7%, sham mean = 53.1%, *p* = 0.883).

### Empirical Analysis

We employed a logit model to examine the effect of tDCS on deceptive behavior in conflict trials. The dependent variable was *Honesty*, which was a dummy variable and equaled one if the sender sent the honest message to receiver, and otherwise zero. As there were three stimulation types, we set a dummy variable *Left* to be one for left anodal/right cathodal tDCS and otherwise zero, and another dummy variable *Right* to be one for right anodal/left cathodal tDCS and otherwise zero. *Sender-interest gap* and *receiver-interest gap* were two variables representing the absolute difference between the payoff of the two options for senders in each trial and the absolute difference between the payoff of the two options for receivers in each trial. We also included *Trial* as a control variable. **Table 2** provided the results of the logit models.

**Table 2:** Regression results for deceptive behavior.

	Full sample
Left	-0.04 (-0.25)
Right	0.52^∗∗∗^ (2.98)
Male	0.45^∗∗∗^ (3.18)
Sender-interest gap	-0.16^∗∗∗^ (-7.62)
Receiver-interest gap	0.12^∗∗∗^ (7.05)
Trial	-0.01 (-0.28)
Pseudo *R*^2^	0.0788
*P*-value	0.0000
Observation	900


*Significance level: ^∗∗∗^ 1 percent, ^∗∗^ 5 percent, ^∗^ 10 percent; *t*-values in parentheses*.

According to the regression results of the full sample, *Right* was significant and its coefficient was positive, while the coefficient of *Left* was not significant. It meant that senders were more likely to send the honest message after the right anodal/left cathodal tDCS over DLPFC which was consistent with the test results in Section “Effect of tDCS on Deceptive Behavior.” The estimated coefficient of *Male* was significant and positive. That is, males were more honest than females. In addition, the significantly negative coefficient of *Sender-interest gap* indicated that the higher the absolute difference between the payoff in the two options for senders, the less likelihood that senders sent the honest message which could decrease self-interest. However, the significant positive coefficient of *Receiver-interest gap* indicated that the higher the absolute difference between the payoff in the two options for receivers, the more likelihood that senders sent the honest message which could increase the interests of others. These findings might provide evidence that deception related to the trade-off between self-interest and others’ interests.

### Questionnaire

To directly learn the sender’s belief, in the questionnaire, we asked senders whether they believed that the receiver would follow their messages. If the answer was no, we further asked them whether they would deliberately send the honest message to mislead the receiver. In fact, only 5.26% (3 of 57) of the senders admitted that they would choose to tell the truth because they expected the receivers not to follow their messages. Therefore, according to the supplementary questionnaire, we can conclude that the senders’ strategic considerations were nearly non-existent in our experiment, and indeed, the transcranial direct current stimulation influenced their deceptive behavior.

## Discussion

The objective of this study was to investigate the effect of modulating the activity of the DLPFC on deception. In the experiment, we used a between-subject design and a cheap talk sender–receiver task from which we were able to measure the honest/dishonest decisions of subjects and uncover the effect of tDCS on deception by comparing different treatments. Direct evidence of a causal relationship between DLPFC and deceptive behavior was provided. We found that modulating the activity of the DLPFC using right anodal/left cathodal tDCS significantly decreased subjects’ deception; they became more honest after right anodal/left cathodal stimulation of the DLPFC. A gender difference in deceptive behavior was also observed. To better learn the sender’s beliefs regarding the receiver’s reaction to messages, we used an additional questionnaire. Only 5.26% senders in the questionnaire would deliberately choose to be honest because they believed receivers would not follow their messages. The results implied that most of the senders did not have strategic considerations.

Conflict between self-interest and others’ interest is of great importance in subjects’ deceptive behavior. Gneezy (2005) defines four categories of lie: (1) white lies, which may be helpful, or at least do no harm to anyone; (2) lies that help others but harm the liar; (3) lies that may not help the liar but harm others or harm both sides; and (4) lies that increase the liar’s payoffs and decrease others’ payoffs. His study showed that people may dislike cheating but will lie for considerable benefits when there is interest conflict. The focus of our paper is the third and fourth categories, especially the fourth, which is relevant to many economic events. In our experiment, deception was ubiquitous in the sham stimulation treatment. Senders are expected to manipulate receivers to improve their own interests and damage receivers’ interests in conflict trials. We found that subjects were significantly more honest in no-conflict trials than in conflict trials. To some extent, it suggests that deception is self-interest driven (Mead et al., 2009).

Our study also demonstrated the important role of DLPFC in modulating self-interested driven deceptive behavior. Importantly, we found that deception could be significantly decreased with right anodal/left cathodal stimulation of the DLPFC, which may help to detecting deception using neurotechnologies. People became more honest after such stimulation in terms of both the percentage of honest choices made and the amount given. Moreover, the effect of tDCS on deceptive behavior was only significant when senders’ own interests were in conflict with receivers’ interests, which was partly in support of the results of Zhu et al. (2014) showing that DLPFC patients behaved differently in conflict trials and no-conflict trials. It is reasonable that modulating the activity in DLPFC will affect subjects’ deceptive behavior, because conflict related deception in our experiment needs the executive control which is an important function of DLPFC (Macdonald et al., 2000). Our research extended the effect of tDCS on deceptive behavior when honest choices were associated with conflict between subjects’ self-interest and others’ interests, which was different from the study by Maréchal et al. (2017), which only considered self-interest and honesty, regardless of others’ benefit.

The gender did make differences in the effect of transcranial direct current stimulation over dorsolateral prefrontal cortex on deception. Experimental economic studies in the literature have shown that gender differences are substantial concerning risk aversion, corruption, competitiveness, as well as deception (Gneezy et al., 2003; Dreber and Johannesson, 2008; Croson and Gneezy, 2009; Frank et al., 2011; Marchewka et al., 2012). In the sham stimulation, we found that gender differences were significant in deceptive behavior and males were more honest than females in conflict trials. This was not consistent with the previous study by Dreber and Johannesson (2008), which replicated the task used by Gneezy (2005) and showed that men were more likely than women to lie for higher amounts of money. One possible explanation for the different findings is that the different monetary allocation between senders and receivers in our experiment compared with Gneezy (2005) might affect senders’ deceptive behavior^1^. We also found that the deceptive behavior of females was significantly decreased after the right anodal/left cathodal stimulation of the DLPFC but the effect was not significant for males. Because the percentage of honest choices was already high for males in the sham stimulation, the small amount of room for improvement in the honesty of males may have resulted in the insignificant effect of tDCS on males’ deceptive behavior.

Two limitations of our study should be noted. One is the problem of the focality of tDCS. Specifically, it is hard to determine whether the observed effects of tDCS were due to selective modulation of the target area or due to the inevitable widespread and non-selective modulation over the cortex (Sellaro et al., 2016). Second, there is a remained question that whether the neural process of conflict related deception in DLPFC is specialized for resolving moral conflicts between self-interest and others’ interest or it is just a general brain response to conflict resolution.

In sum, our results suggest that the neural basis of deception is mainly managed by the activity of the DLPFC. Modulating the activity of the DLPFC using right anodal/left cathodal tDCS significantly decreased subjects’ deception. Honesty is very important in economic and social relationships, so it is meaningful to explore its neural process to have a better understanding of the basis of people’s deceptive behavior. We may design other deception games including four kinds of lies defined by Gneezy (2005) to investigate the effects of tDCS over DLPFC on deception with different interest conflicts, and we should also add moral attitude measurement and other conflict related task to the experimental design to better understand the neural mechanism of deception in further study.

## Author Contributions

XY, MG, and YL designed the experiments. MG and YL performed the experiments. SC and MG analyzed the data. SC and YL drew the figures. XY, MG, JS, YL, and SC wrote the manuscript, revised the manuscript, and finally approved the version to be published.

## Conflict of Interest Statement

The authors declare that the research was conducted in the absence of any commercial or financial relationships that could be construed as a potential conflict of interest. The reviewer HW and handling Editor declared their shared affiliation.

## Footnotes

1 There might also be other factors making the result uncertain. Gylfason et al. (2013) used the Gneezy (2005)’s design but found no significant gender differences.
